# Full-surface emission of graphene-based vertical-type organic light-emitting transistors with high on/off contrast ratios and enhanced efficiencies

**DOI:** 10.1038/s41598-019-42800-y

**Published:** 2019-04-19

**Authors:** Byoungchoo Park, Won Seok Lee, Seo Yeong Na, Jun Nyeong Huh, In-Gon Bae

**Affiliations:** Department of Electrical and Biological Physics, Kwangwoon University, Wolgye-Dong, Nowon-gu, Seoul, 01897 Republic of Korea

**Keywords:** Electronic devices, Organic LEDs, Electronics, photonics and device physics, Optical properties and devices, Electronic properties and devices

## Abstract

Surface-emitting organic light-emitting transistors (OLETs) could well be a core element in the next generation of active-matrix (AM) displays. We report some of the key characteristics of graphene-based vertical-type organic light-emitting transistors (Gr-VOLETs) composed of a single-layer graphene source and an emissive channel layer. It is shown that FeCl_3_ doping of the graphene source results in a significant improvement in the device performance of Gr-VOLETs. Using the FeCl_3_-doped graphene source, it is demonstrated that the full-surface electroluminescent emission of the Gr-VOLET can be effectively modulated by gate voltages with high luminance on/off ratios (~10^4^). Current efficiencies are also observed to be much higher than those of control organic light-emitting diodes (OLEDs), even at high luminance levels exceeding 500 cd/m^2^. Moreover, we propose an operating mechanism to explain the improvements in the device performance *i.e*., the effective gate-bias-induced modulation of the hole tunnelling injection at the doped graphene source electrode. Despite its inherently simple structure, our study highlights the significant improvement in the device performance of OLETs offered by the FeCl_3_-doped graphene source electrode.

## Introduction

Organic light-emitting transistors (OLETs) have been developed by integrating the ability of organic light-emitting diodes (OLEDs)^1–6^ to generate light with the electrical-switching functionality of organic field-effect transistors (FETs)^7,8^ into a single device structure^9–29^. In OLETs, the current flowing through emissive semiconductor channel layers can be modulated by the gate voltage, which can also change the electroluminescent (EL) emission brightness from the dark off-state to the bright on-state^9–29^. In addition to such EL switching functionality, OLETs have other advantages over OLEDs, such as relatively high luminance efficiency and external quantum efficiency^13,14^. Thus, OLETs are of key interest, as they can provide a unique type of device architecture for investigating fundamental opto-electronic properties related to the charge carrier injection, transport, and radiative exciton recombinations in organic semiconducting materials. Moreover, due to their compact architecture, they can be used to develop integrated organic opto-electronic devices such as highly efficient light sources, optical communication systems, and/or electrically driven organic lasers^11–18^.

For these principal reasons, the luminance from an OLET can be modulated without any additional driving devices, and displays using OLETs thus offer the additional advantage of reducing the number of high mobility-driving thin film transistors (TFTs) and simplifying the inherent complexity of the circuits of conventional active-matrix (AM) OLEDs^14,17,20^. In this sense, OLETs can be an effective means of increasing the aperture ratio (the light-emitting area as a proportion of the total area of the device), making it higher than that (~34%) of a typical AM-OLED^17,20^. Hence, surface-emitting OLETs may offer an attractive alternative to conventional AM displays as a key element in the development of next-generation AM display technology^17,20^.

Most OLETs have lateral source-drain geometries but exhibit line-, band-, or circular-type emission characteristics in a limited zone between the source and drain electrodes^9–20^. Thus, much effort has been made to improve the device performance and to extend the light emission area; multilayer structures^12,14,16^ and/or modified electrodes^18–20^ have been suggested to control the charge injection, charge transport, and charge carrier recombinations in the emissive channel layers of these devices.

To obtain highly increased source drain current flows even at low gate voltages, vertical OLETs (VOLETs) were also constructed with short channel lengths by coupling a static induction transistor structure^21,22^, constructing a metal insulator semiconductor structure^23,24^, or using micro-networks with periodic vacancies in a vertical configuration^25,26^. These devices have shown stripe-type or quasi-surface emission patterns. In these cases, a reliable and high-resolution patterning method for electrodes and/or an insulating charge-restriction layer would be crucial to control the leakage currents and switching characteristics.

In another effort, a novel type of VOLET and a related fully functional display were developed using a source electrode consisting of randomly distributed carbon nanotubes (CNTs)^27–29^. The CNT-based VOLET (CNT-VOLET) has shown a number of remarkable improvements, such as highly bright and efficient full-surface emissions with high on/off ratios^27,28^. This good switching ability was mainly attributed to the gate-voltage-induced modulation of the lateral (or horizontal) Schottky barrier height for the dilute network of the CNT source electrode in the CNT-VOLET^27,28^. Nevertheless, the CNT-VOLET may experience non-uniformity as well as poor connectivity of the CNTs in the source electrode^8,25,26^. In addition, the irregular interface between the CNT source electrode and its adjacent functional layer may act as traps to hinder the charge transport^25,26^. Moreover, the CNT electrode typically forms a very rough surface, thus requiring a fairly thick adjacent functional layer^8,28^. Thus, the ultimate goal of simple and reliable OLETs capable of high device performance with good switching ability remains unmet.

In this article, we report on the first use of a VOLET with a homogeneous, smooth, and easily processable graphene layer as the source electrode, together with an emissive channel layer. As a two-dimensional material in the form of a single layer with a carbon-based hexagonal lattice structure bonded in the sp^2^ configuration^30–32^, graphene has been used as a transparent electrode material in small OLEDs, as a proof of concept^33–35^. Despite the low dimensionality of graphene, similar to that of CNTs^31,32^, the successful operation of full-surface EL light emission from a VOLET based on graphene has not yet been reported, as the work function (~4.6 eV) of pristine graphene is too low for the hole injection^33–35^. We describe the fabrication and characterization of a simple graphene-based VOLET (Gr-VOLET) with a FeCl_3_-doped single-layer graphene (SLG) source electrode. We find that the EL properties of the Gr-VOLET can be efficiently modulated with high luminance on/off ratios (~10^4^) through the application of gate voltage. More interestingly, our Gr-VOLETs with doped SLG sources are shown to exhibit greatly improved device performance, especially for their higher current efficiencies as compared to those of control OLEDs, even at high luminance levels exceeding 500 cd/m^2^, making them all the more attractive. We discuss the operating mechanism that explains these significant improvements in the device performance; *i.e*., the effective modulation of the hole tunnelling injection from the FeCl_3_-doped graphene source.

## Results and Discussion

### Operating characteristics of Gr-VOLETs

**Figure 1:**
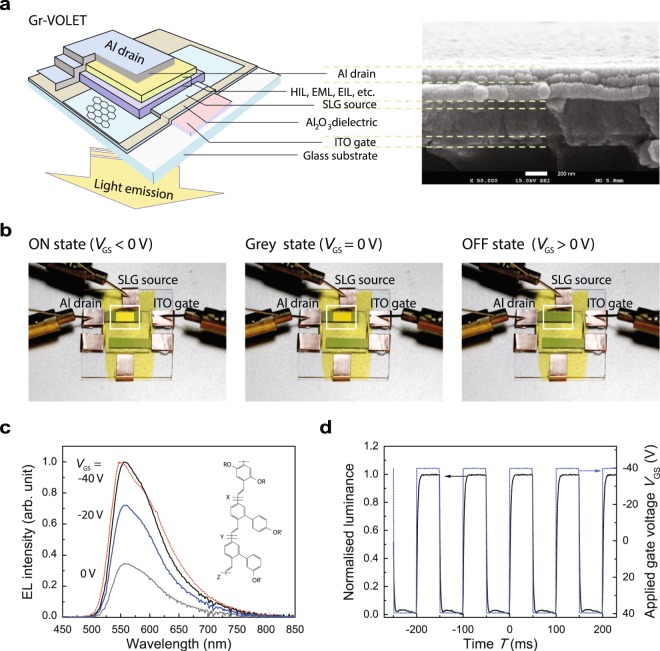
Structure and operation of graphene-based vertical organic light-emitting transistors (Gr-VOLETs). (**a**) Schematic illustration of the structure and a SEM image of a cross-sectional slice of the Gr-VOLET with a single-layer graphene (SLG) source, stacked layers of organic functional channel layers, an Al drain, and an ITO gate separated with an Al_2_O_3_ gate dielectric (scale bar: 200 nm). (**b**) Light emission from a Gr-VOLET (4 mm × 2 mm, white squares) for three different gate voltages of *V*_GS_ for a fixed source-drain voltage, *V*_SD_, of 3.8 V (see also Supplementary Video S1 and Supplementary Fig. S1b). (**c**) Relative electroluminescent spectra of a Gr-VOLET (solid curves) and of a corresponding control ITO-OLED (dotted curve). The molecular structure of Super Yellow (SY) is shown in the inset. (**d**) Temporal response of a Gr-VOLET with respect to the step gate voltages for a fixed *V*_SD_ of 3.8 V.

Our first challenge relates to the structure and operating characteristics of our Gr-VOLET, including the bottom indium tin oxide (ITO) gate, the Al_2_O_3_ gate dielectric layer, the SLG source, the functional channel layers including the organic light-emitting layer (EML) of the Super Yellow (SY) conjugated copolymer, and the Al metal drain, in sequence (Fig. 1a, see also Methods and Supplementary Fig. S1a for details of the structure and layer thicknesses). During the operation of the Gr-VOLET, the electron injection occurs from the Al drain into the channel layer, and the hole injection from the SLG source can be modulated by adjusting the gate voltage *V*_GS_, as discussed below. Figure 1b shows the EL light emissions of a sample Gr-VOLET operating under different *V*_GS_ levels with a fixed source-drain voltage *V*_SD_ of 3.8 V. As shown in the figure, the EL light emission is uniformly bright (in the fully on-state), grey, and dark (off-state) over the entire surface of the active area for negative, zero, and positive *V*_GS_ values, respectively. (See also Supplementary Information Video 1 and Supplementary Fig. S1b for images of the device in the dark). Hence, *V*_GS_ essentially influences the hole injection and current flow through the emissive channel layer and thus may contribute to the charge balance for efficient hole-electron recombinations in the channel layers. The EL emission spectra observed are nearly identical to those obtained from a conventional ITO-OLED (Fig. 1c). Furthermore, the temporal responses of the Gr-VOLET with respect to step changes in *V*_G_ show rapid rising and falling times of 4.7 ms and 2.8 ms, respectively (Fig. 1d), which are also comparable to those of conventional OLEDs and much faster than those of traditional liquid crystal displays (LCDs)^5^. The above characteristics highlight several interesting features of our Gr-VOLET. To investigate its distinct characteristics further, we tested three types of SLG materials as source electrodes; (i) p-doped SLG with FeCl_3_ (hereafter SLG_1_ for fabricating Gr-VOLET_1_), where FeCl_3_ doping is done spontaneously during the graphene transfer process, as shown previously^35^; (ii) pristine (intrinsic) SLG, cleaned by an electrochemical process^35^, as a comparative reference (SLG_2_ for Gr-VOLET_2_); and (iii) SLG coated with a conventional hole-injection layer (HIL) of (3,4-ethylenedioxythiophene):poly(styrene sulfonate) (PEDOT:PSS)^34^ as a second comparative reference (SLG_3_ for Gr-VOLET_3_). The basic properties of the three SLG sources (with low porosities below 0.1%) are shown in Supplementary Fig. S2,S3 and S4 and are summarised in Table 1.

**Table 1:** Summary of the basic electronic properties of the SLGs studied here.

SLGs	Sheet resistance, (kΩ/square)	Work function* (eV)	Dirac point energy* (eV)	Hole/electron mobility (cm^2^/(V s))
SLG_1_	1.20	5.21 ± 0.07	4.89	410/−
SLG_2_	2.40	4.70 ± 0.10	4.44	580/530
SLG_3_	1.40	5.21 ± 0.06	4.98	530/−

Average values were obtained from five individual devices for each of the device configurations studied.

**Figure 2:**
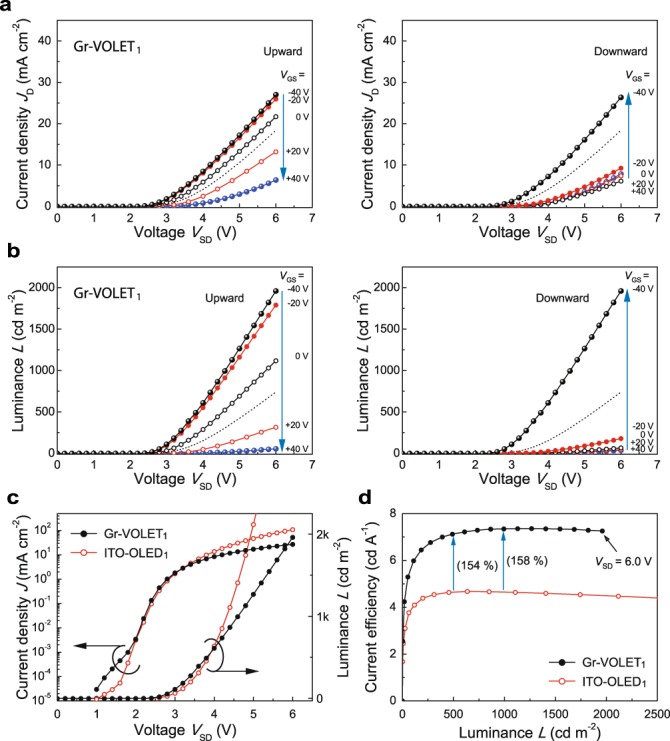
Output characteristics of Gr-VOLET_1_ and comparison with the control ITO-OLED. Gate-voltage (*V*_GS_)-dependent current density-voltage (*J*_D_*-V*_SD_) (**a**) and luminance-voltage (*L-V*_SD_) (**b**) characteristics of Gr-VOLET_1_ with a FeCl_3_-doped SLG_1_ source for upward (left) and downward (right) changes in *V*_GS_. For comparison, the characteristics of a gate-disconnected Gr-VOLET_1_ (*i*.*e*., Gr-OLED_1_) are also shown (dotted curves). *J-L-V* (**c**) and *η*_C_-*L* (**d**) comparisons of Gr-VOLET_1_ in the bright on-state (*V*_GS_ = −40 V) with its respective ITO-based control OLED (ITO-OLED_1_, ITO/SY/CsF/Al).

We now describe the detailed output current and luminance characteristics of the three prototype Gr-VOLETs mentioned above. For comparative purposes, we also observed the diode characteristics of the Gr-VOLETs with the gate electrodes disconnected from the external circuits (Gr-OLEDs) (Fig. 2a and Supplementary Figs S5a and S6a). As shown, the current density-voltage (*J*_D_*-V*_SD_) and luminance-voltage (*L-V*_SD_) characteristics of the Gr-VOLETs present four key characteristics: (1) the *J*_D_*-V*_SD_ characteristics are similar to those of a diode without current saturation, which were generally observed in vertical-type organic field-effect transistors due to the short vertical channel lengths^8^, (2) similar behaviours to the *J*_D_*-V*_SD_ curves were observed in the *L-V*_SD_ characteristics, (3) both *J*_D_ and *L* for a given *V*_SD_ increase with a decrease in the negative *V*_GS_, even at a low *V*_SD_, which shows that current modulation by *V*_GS_ can change EL emission brightness. Hence, the *V*_GS_-dependent turn-on voltage (*V*_onset_) can be reduced to well below *V*_onset_ of the Gr-OLED, and (4) both *J*_D_ and *L* also depend on the direction of the change of *V*_GS_, *i.e*., increasing (upward)/decreasing (downward), implying hysteretic behaviour. Among the Gr-VOLETs, interestingly, the Gr-VOLET device with the doped SLG_1_ source (Gr-VOLET_1_, Fig. 2a) exhibits highly improved device performance, superior to that of Gr-OLED_1_. For example, at *V*_GS_ = −40 V, the *J*_D_ value is higher than that of Gr-OLED_1_ and the luminance reaches *L* ~ 2,000 cd/m^2^ at *V*_SD_ = 6.0 V (*V*_onset_ = 2.3 V), which is more than twice that (*L* ~ 740 cd/m^2^ and *V*_onset_ = 2.5 V) of Gr-OLED_1_. These outcomes indicate improved and balanced charge (hole) injections from the SLG_1_ source in the case of a negative *V*_GS_. Conversely, at *V*_GS_ = +40 V, *J*_D_ and *L* of Gr-VOLET_1_ are much lower, possibly due to the switching off of the hole injection from the SLG source. The highest values observed for the peak on/off ratios of *J*_D_ and *L* were approximately 10^2^ and 10^4^, respectively, at *V*_GS_ = ± 40 V. This gate-bias-induced modulation effect of Gr-VOLET_1_ is shown to be more efficient than those of the other Gr-VOLETs tested here with the pristine (pure) SLG_2_ source (Gr-VOLET_2_), or even with the PEDOT:PSS HIL-coated SLG_3_ source (Gr-VOLET_3_).

We then estimated the device performance capabilities of the Gr-VOLETs in the on-state (*V*_GS_ = −40 V), comparing these with control OLEDs fabricated using the same batch process on ITO anodes (ITO-OLEDs) (Fig. 2b and Supplementary Figs S5b and S6b). As shown, only Gr-VOLET_1_ exhibits luminance higher than that of the control ITO-OLED (ITO/SY/CsF/Al) in the source-drain voltage region *V*_SD_ < 4.0 V (Fig. 2b). For example, when *V*_SD_ = 3.8 V, Gr-VOLET_1_ emitted luminance of 490 cd/m^2^, while the control ITO-OLED emitted luminance of 455 cd/m^2^ at *V* = 3.8 V. Moreover, Gr-VOLET_1_ was found to be more efficient than the control ITO-OLED, in contrast to the other Gr-VOLETs (Fig. 2c and Supplementary Figs S5c and S6c). For instance, at a luminance level of 500 cd/m^2^, Gr-VOLET_1_ emitted EL light with current efficiency *η*_C_ of 7.13 cd/A, which is approximately 1.54 times higher than that (4.64 cd/A) of the control ITO-OLED (*η*_C Gr-OLET_/*η*_C OLED_ = 1.54). Even at luminance levels exceeding 2,000 cd/m^2^, Gr-VOLET_1_ maintains an enhanced *η*_C Gr-OLET_/*η*_C OLED_ ratio of ~1.62. It is therefore clear that Gr-VOLET_1_ has highly enhanced current efficiency compared to other devices investigated (Table 2). This offers another important advantage: given this level of enhanced current efficiency, the brightness of the device can be maintained with a lower *J*_D_, promising a longer device lifetime^6^. It is noteworthy that *η*_C_ (7.13 cd/A) for Gr-VOLET_1_ was approximately 1.38 times higher than that (5.17 cd/A) of ITO-OLED_3_, the latter possessing the optimised HIL of PEDOT:PSS (Supplementary Fig. S6c). Thus, it is clear that the SLG_1_ source in Gr-VOLET_1_ provides amplification of both the emission and current efficiency, although further optimization of the drain electrodes is still possible.

**Table 2:** Comparison of the current efficiencies of Gr-VOLETs (*η*_C Gr-OLET_) and ITO-based control OLEDs (*η*_C OLED_) with their *η*_C Gr-OLET_/*η*_C OLED_ ratios at a luminance level of 500 cd/m^2^.

Devices	*η*_C Gr-OLET_ (cd/A)	*η*_C OLED_ (cd/A)	*η*_C Gr-OLET_/*η*_C OLED_^a^
Gr-VOLET_1_	7.13	4.64	1.54
Gr-VOLET_2_	2.92	4.64	0.63
Gr-VOLET_3_	3.72	5.17	0.72

^a^Ratio of the current efficiency of a full-surface emitting Gr-OLET to that of the control ITO-based OLED for a given luminance level of 500 cd/m^2^.

### Charge injection process at SLG sources

**Figure 3:**
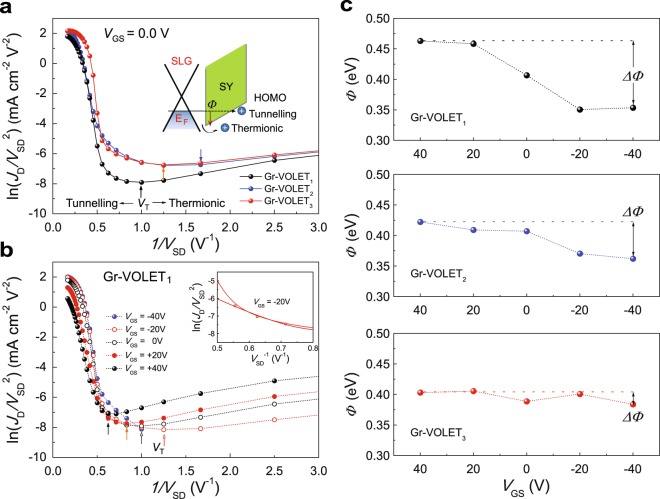
Charge injection processes in Gr-VOLETs. Fowler-Nordheim plot, ln(*J*_D_/*V*_SD_^2^) vs 1/*V*_SD_, for Gr-VOLETs with different SLG sources at *V*_GS_ = 0 V (**a**) and with the SLG_1_ source at various *V*_GS_ levels (**b**) for upward *V*_GS_ changes. The inset in (**a**) shows a schematic energy band diagram of the thermionic emission and tunnelling at the SLG/SY interface along the normal direction of the interface between the SLG and the SY channel layer. The inset in (**b**) shows an example of the theoretical fittings based on the tunnelling current model (solid curve). (**c**) Gate-bias-modulated hole tunnelling barrier height, *Φ*, extracted from the fittings in the hole-dominant regimes. *ΔΦ*: gate-bias-induced *Φ* modulation when *V*_GS_ =  ± 40 V.

Our investigation also focused on the hole injection mechanism from the SLG source into the SY channel layer. To be injected across the SLG/SY interface, the holes must overcome the potential barrier at the interface either via thermionic emission or tunnelling processes^36–41^. Figure 3a shows examples of Fowler-Nordheim (F-N) plots^36–41^, ln(*J*_D_/*V*_SD_^2^) vs 1/*V*_SD_, for the three Gr-VOLETs when *V*_GS_ = 0 V during upward changes in *V*_GS_. All of the plots show two distinct hole injection processes with the transition voltage (*V*_T_), at which value the injection mechanism changes from Schottky thermionic emission to tunnelling^39–41^.

Figure 3b presents F-N plots of a typical Gr-VOLET_1_ at various values of *V*_GS_ during upward *V*_GS_ changes. It is interesting to note that *V*_GS_ affects both Schottky thermionic emission and tunnelling; thus, *V*_T_ strongly depends on *V*_GS_. It is also noteworthy that because the EL emission from the Gr-VOLETs occurs when *V*_SD_ > *V*_onset_, higher than *V*_T_, the main hole injection process for light emission is tunnelling injection in the Gr-VOLETs. According to a modified tunnelling current model^42^, the tunnelling current density (*J*) for a single charge carrier through a triangular barrier at a metal/polymer junction is related to the potential barrier height *Φ* and the temperature *T*: ln(*J*/*V*^2^) = −*P*_1_/*V* + ln(*P*_2_/*V*) − ln[sin(*P*_3_/*V*)], with *Φ* = (3/2) π*k*_B_*T* (*P*_1_/*P*_3_), where *k*_B_ is the Boltzmann constant, and *P*_i_ denotes parameters related to *Φ*^42^. This relationship allows the F-N curves to be analysed, and the potential barrier heights *Φ* at the SLG/SY interfaces between the Fermi level of the SLGs and the highest occupied molecular orbital (HOMO) level (~5.3 eV) of the SY channel layer^35^ to be obtained (Fig. 3c), all of which are in reasonable agreement with values in the literature^43^. Among the SLG/SY interfaces, the SLG_1_/SY interface exhibited the strongest gate-bias-induced *Φ* modulation (*ΔΦ*) along the direction normal to the interface; *i*.*e*., *ΔΦ* at *V*_G_ = ±40 V was approximately 110 meV, which is much higher than the values of *ΔΦ* for SLG_2_/SY (~60 meV) and SLG_3_/SY (~20 meV). This strongest gate-bias-induced *Φ* modulation of the FeCl_3_-doped SLG_1_ source leads to the efficient modulation of the device performance of Gr-VOLET_1_ as tested here. Note that when *V*_SD_ > *V*_onset_, the theoretical predictions begin to deviate from the experimental data, mainly due to minority carrier (electron) injections into the SY channel layer from the Al drain. Nevertheless, tunnelling at the SLG/SY interface is the major hole injection process, being responsible for the radiative recombination of electron-hole pairs. This analysis is also supported by an inspection of the weak dependence on *T* of *J*_D_-*V*_SD_ and *V*_T_ for Gr-VOLET_1_ (Supplementary Fig. S7); the other injection process, Schottky thermionic injection, is in contrast strongly dependent on *T*^36,39,40^. Therefore, the tunnelling analysis provides clear evidence that our device operates via the modulation of the vertical barrier height along the direction normal to the source surface (*i*.*e*., parallel to the gate field direction), in contrast to the CNT-VOLET based on lateral (or horizontal) Schottky barrier height modulation along the horizontal direction on the source surface (*i*.*e*., perpendicular to the gate field direction)^27,28^, and different as well from conventional graphene-based barristors that operate via the modulation of the Schottky thermionic injection^44^. It is also noted that if one considers the further contribution of the gate field to the electric field inside the channel layer together with an appropriate dielectric constant of the channel layer, one may then obtain a more precise value of the barrier height from the F-N analysis for the injection characteristics. However, this is beyond the scope of this report, and further details about such an analysis and a related discussion will therefore be reported elsewhere.

**Figure 4:**
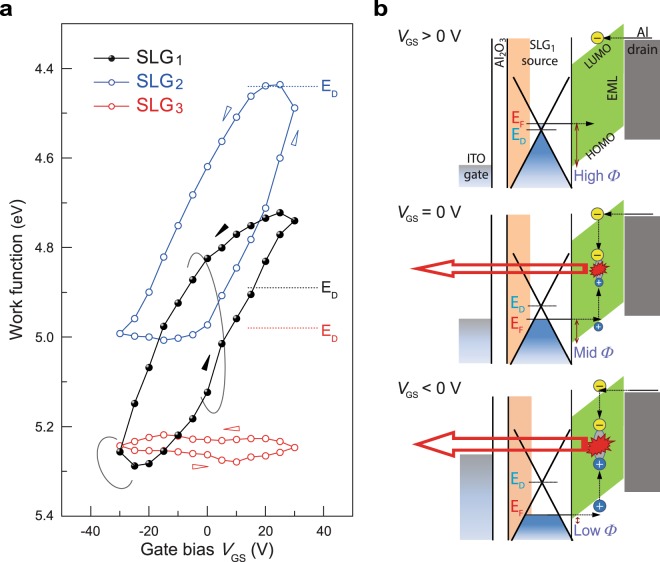
Gate-bias-induced modulation of SLG work functions and operation mechanism of Gr-VOLETs. (**a**) Gate-bias-induced modulation of the work functions of SLG sources on the VOLET substrates. (**b**) Energy-level diagrams of Gr-VOLET_1_ for high, mid, and low *Φ*s at three distinct values of *V*_GS_ at a given *V*_SD_. *Φ* depicts the tunnelling barrier height for the hole injection. *E*_D_: Dirac point energy of the SLG source used.

To gain a better understanding of the hole injection mechanism at the SLG sources, we also investigated the dependence of the Fermi levels (work functions) of the SLG sources on the gate bias using the KPFM method (see also Supplementary Fig. S3). We observe that a *V*_G_ sweep clearly modulates the work functions of the SLGs. As shown in Fig. 4a, for the pristine SLG (SLG_2_), a large change in the work function can be observed, from 4.44 eV to 5.00 eV, by sweeping *V*_GS_. However, for the doped SLG (SLG_1_), a largely downwardly shifted modulation of the work function was observed, from 4.72 eV to 5.29 eV. This work function of SLG_1_ is closer to the HOMO level of the SY channel layer than that of SLG_2_ at a negative gate bias. In contrast, the PEDOT:PSS-coated SLG (SLG_3_) shows only a minor modulation of the work function. This may be due to the negatively charged PSS in the PEDOT:PSS HIL on the SLG_3_ source^45^, which can generate a strong electrostatic field and accordingly weaken the gate-field effect, as also shown in Supplementary Fig. S6a. In addition, considerable hysteretic behaviour is apparent in all of the plots, possibly due to charge trapping in the Al_2_O_3_ dielectric layer^46^. These hysteretic work functions of SLGs are major causes of the hysteresis of the output characteristics of the Gr-VOLETs for upward and downward *V*_GS_ changes.

The above observations reveal the working principle of Gr-VOLET_1_, as illustrated in the energy-level diagrams in Fig. 4b. At a given *V*_SD_, a positive gate bias induces an upward shift of the Fermi level of the SLG_1_ source in a direction that increases the barrier height *Φ*, resulting in reduced tunnelling and fewer hole injections into the HOMO level of the SY channel layer. In contrast, a negative gate bias induces a downward shift of the Fermi level of the SLG_1_ source, decreasing *Φ* significantly (enhancing tunnelling) and hence allowing increased hole injection and improved EL performance. Thus, together with the band-bending effect^27^, the main operating mechanism of Gr-VOLET_1_ is energy band matching, and the charge balance is thus achieved even without any HIL through gate-bias-induced modulation of the hole tunnelling injection at the SLG source, as controlled by p-type doping with FeCl_3_. Although the work function of SLG_3_ including the PEDOT:PSS HIL is nearly identical to that of SLG_1_, the modulation performance of Gr-VOLET_3_ is considerably lower than that of Gr-VOLET_1_. This may arise when the PEDOT:PSS HIL impedes the effect of *V*_G_ on the modulation of the barrier height *Φ* due to its strong electrostatic field, in conjunction with the gate field-screening effect in the highly conductive PEDOT:PSS layer. Note that the changes in the potential barrier height *Φ* induced by *V*_GS_ are somewhat lower than the changes in the work functions of the SLGs caused by *V*_GS_, which may be due to the field effect of *V*_SD_ applied to the SLG sources together with the electrostatic interaction effect via the charge transfer^47^ at the SLG/SY interface (further details will be reported elsewhere).

### Transfer characteristics and TFT-switching Gr-VOLETs

**Figure 5:**
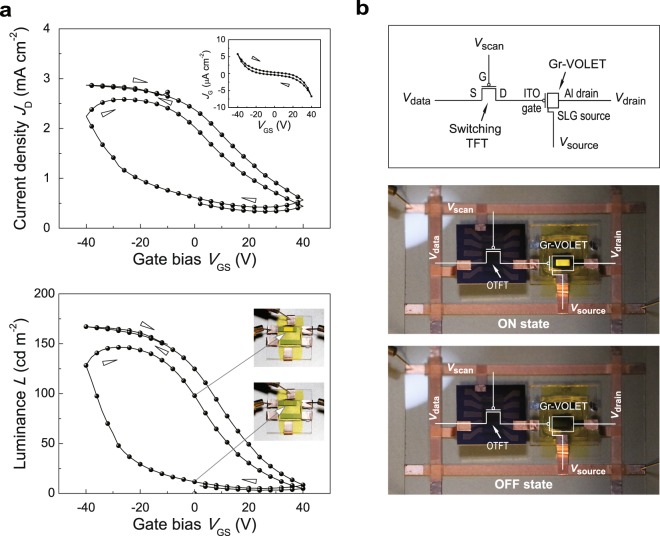
Transfer characteristics and Gr-VOLET switched by an OTFT. (**a**) Transfer *J*_D_*-V*_GS_ (upper) and *L-V*_GS_ (lower) curves of Gr-VOLET_1_ at a given *V*_SD_ of 3.2 V. The insets in the upper and lower panels respectively show the *J*_G_*-V*_GS_ characteristics of Gr-VOLET_1_ and two photographs of the Gr-VOLET at *V*_GS_ = 0 V for a given *V*_SD_, revealing bistable-like operation. (**b**) A simple AM-OLET pixel circuit diagram of a Gr-VOLET combined with a switching TFT (upper), and two photographs of Gr-VOLET_1_ switched by an OTFT, showing the bright on-state (middle) and the dark off-state (lower).

We now turn our attention to the transfer characteristics of Gr-VOLET_1_. As shown in Fig. 5a, at a given *V*_SD_ (3.2 V), *J*_D_ increases when *V*_GS_ is biased towards higher negative values, and thus Gr-VOLET_1_ is normally in the “on-state,” while it is “switched off” at a positive gate bias. During the switching operation, the device exhibits a fairly low gate leakage current density (*J*_G_) (inset in the upper panel). Similar to the output curves, no region of saturation is observed in the transfer curves for the *V*_G_ range applied. In addition, notable instances of hysteresis are clearly observed in the transfer curves, caused by the hysteretic loop of the work function of the SLG_1_ source, as shown above; bistable-like switching operations (or memory-like effects) of Gr-VOLET_1_ are thus verified at *V*_GS_ = 0 V, as shown in the insets in the lower panel. This property of a Gr-VOLET may allow novel applications for inexpensive and simple driving schemes with low power consumption. However, this effect may become more significant when realising high-quality grayscale; moreover, it should be carefully controlled when preparing the dielectric layer. It is also noted, as shown in the figure, that the on/off ratios of the current and luminance modulations are somewhat reduced, mainly due to the increased leakage current between the source and drain via the deterioration of the device during the measurement process. Thus, to maintain the high on/off ratios of the devices, one should control the degradation and leakage current flows precisely.

To investigate its switching capability, we now describe an application of our Gr-VOLET combined with only one switching device, excluding any other high mobility-driving TFTs or storage capacitors as commonly used in conventional AM-OLEDs (Fig. 5b)^4^. Here, the switching device used was an organic TFT (OTFT), produced using 6,13-bis(triisopropylsilylethynyl)pentacene blended with a polymer binder of poly(a-methylstyrene), with mobility of approximately 0.1 cm^2^/(V s) (see Supplementary Fig. S8). For its operation, a *V*_DS_ value of ±80 V and a *V*_GS_ value of −50 V were applied to the OTFT, resulting in *V*_GS_ applied to the gate of the Gr-VOLET being as high as ±20 V with a fixed *V*_SD_ of 3.2 V for the Gr-VOLET. Most interestingly, the EL light output of the Gr-VOLET_1_ was successfully switched from the bright on-state (middle panel) to the dark off-state (lower panel) by the OTFT operation, despite the fact that the mobility of the OTFT is much lower than that (0.6–1 cm^2^/(V s)) of a typical Si TFT^4^. From this application, we contend that our Gr-VOLET shows considerable promise for reliable and high-performance AM displays.

The foregoing results clearly demonstrate the remarkable device performance of the Gr-VOLET with a FeCl_3_-doped SLG source electrode, showing considerable promise with regard to the development of high-performance OLETs. To the best of our knowledge, this is the first demonstration of high-performance VOLETs fabricated with single-layer graphene source electrodes modified by FeCl_3_ doping, exhibiting high on/off contrast ratios and enhanced efficiency levels.

Finally, two points should be discussed with regard to our Gr-VOLET, the first of which relates to the improvement in its performance and lifetime. The light-emitting performance and lifetime can be improved further through the additional optimization of the materials used^48^. Specifically, in place of SY used as the light-emitting material, it would be possible to use other new materials^3^, including red, green, and blue light-emitting fluorescent or phosphorescent materials, which exhibit much higher brightness levels, efficiencies, and lifetimes than those of the Gr-VOLETs studied here. This could yield very bright and efficient Gr-VOLETs with long lifetimes. Second, it is possible to use a thin dielectric layer grown by other deposition methods, such as atomic layer deposition^49^, rather than the thick Al_2_O_3_ dielectric layer used here. This could yield efficient Gr-VOLETs operating at a low *V*_GS_ levels, *i.e*., below 5 V, enabling the adoption of a-Si TFT backplanes.

The advances afforded by the Gr-VOLET with its reliable switching performance, shown here even at high luminance levels, clearly demonstrate its effective light-emitting transistor functionality and make it a viable candidate for use in new voltage-driving light-emitting devices and highly integrated organic opto-electronics. Further, the combination of these Gr-VOLETs with TFT backplanes will certainly presage the development of inexpensive, fast, large-area, and high-performance AM display devices.

## Conclusions

In summary, we have herein explored the characteristics of a graphene-based VOLET consisting of a homogeneous SLG source, an emissive channel layer, and an Al drain, allowing efficient switching of the device performance with variations of the gate voltage. We have verified that low-drain-voltage operations and increased brightness levels with a high luminance on/off ratio of ~10^4^ can be achieved even without a HIL using a p-doped SLG source with FeCl_3_. Moreover, the current efficiency of the Gr-VOLET was at least 1.5 times higher than that of a control ITO-based OLED at a given luminance level. These significant device performance improvements can be attributed to the efficient modulation of the hole tunnelling injection via gate-bias-induced Fermi-level control of the FeCl_3_-doped SLG source. Together with its simple structure and easy processability, this surface-emissive device using doped graphene provides a new platform for the development of advanced light-emitting devices and/or next-generation emissive display devices.

## Methods

### Preparation of substrates

The transparent VOLET substrate was prepared with a pre-patterned back gate electrode consisting of 80-nm-thick ITO (30 ohm/square sheet resistance) on a glass substrate, with a sputter-deposited aluminium oxide (Al_2_O_3_, 400 nm) top layer as a gate dielectric layer over the ITO gate electrode (glass/ITO/Al_2_O_3_). The VOLET substrate used was pre-cleaned with alcohol, followed by a UV treatment for 5 min immediately prior to the fabrication of the graphene-based devices.

The lateral FET substrate was prepared using a VOLET substrate or a 300-nm-thick layer of thermally grown SiO_2_ as the gate dielectric on a heavily doped n-type (100) Si wafer substrate (0.05 ohm cm) for the OTFT, together with laterally patterned metal source and drain electrodes of a 5.5-nm-thick Cr layer and a 50-nm-thick Au layer formed on the substrate via a conventional vacuum deposition process with a shield mask. The channel length (*L*) and width (*W*) of the FET were 50 μm and 1600 μm, respectively (see Supplementary Fig. S4).

### Transfer of graphene

The procedure used for transferring the chemical-vapour-deposition (CVD)-grown graphene onto a target substrate^35,50^, in this case a VOLET substrate, a FET substrate, or a glass substrate, is described below. The first step involves CVD growth of monolayer graphene on a copper (Cu) foil^35,50^. A clean copper foil was placed in a quartz tube chamber and the temperature was increased to 1000 °C under Ar (10 sccm). For the growth of graphene, a gas mixture of CH_4_ (30 sccm) and H_2_ (10 sccm) was used at ~2.7 × 10^−2^ Pa. The second step involved spin-coating a poly(methyl methacrylate) (PMMA) solution (950PMMAC4, MicroChem) onto the CVD-grown graphene on the copper foil at 3000 rpm for 60 s. The graphene film grown on the back side of the copper foil was then removed by atmospheric-pressure oxygen plasma. Subsequently, a PMMA-coated Cu/Gr (Cu/Gr/PMMA) block with a width of 4 mm and length of 20 mm was floated on an aqueous FeCl_3_ solution (UN2582, Transene Co. Inc.) used to etch the copper foil entirely at 50 °C for 10 min. Subsequently, floating of the PMMA-coated Gr (Gr/PMMA) block on the FeCl_3_ solution was maintained for a further 10 min to dope the Gr film with FeCl_3_, as described previously^35^. Next, the Gr/PMMA block was rinsed with deionised (DI) water several times (10 min) and transferred onto the target substrate, after which the SLG-transferred substrate was dried under reduced pressure (~1 Pa) for 1 h and left in air for one day. The PMMA supporting layer was then removed by dissolving the PMMA in chloroform (60 min), monochlorobenzene (30 min) and chloroform again (30 min) in sequence.

### Cleaning and de-doping of SLGs

For cleaning and de-doping of the SLGs on the substrate, a bubble-free electrochemical (EC) treatment was carried out in a non-aqueous electrolyte of acetonitrile (ACN, 99.8%, Aldrich) with 100 mM of tetrabutylammonium hexafluorophosphate (TBAPF_6_, >99.0%, Aldrich) using a potentiostat (DY2113, Digi-Ivy, Inc.)^35^. The SLG transferred onto the substrate was used as a working electrode with a platinum wire as a counter electrode and an Ag/AgCl electrode (3.5 M KCl) as a reference electrode^35^.

The EC-cleaning treatment was conducted using freshly prepared SLG under negative voltage ranges (0.0 ~ −0.7 V/V_Ag/AgCl_) at a voltage-sweeping rate of 0.5 V/s for 10 min. After the cleaning process, the treated SLG was rinsed several times using pure ACN and DI water and then dried with N_2_ gas to remove the electrolyte entirely from the SLG surface. To calibrate the electrode potentials, ferrocene (98%, Sigma Aldrich) was used as a redox probe.

### Fabrication of VOLETs

The fabrication steps of the Gr-VOLET used in this study are described below (see Supplementary Fig. S1a). To construct the Gr-VOLET, SLG with an area of 4 mm by 20 mm was transferred onto the VOLET substrate, as mentioned above. The SLG electrodes used were FeCl_3_-doped SLG (SLG_1_) or EC-cleaned (pristine) SLG (SLG_2_). Next, organic semiconducting materials were deposited over the source electrode regions; a 70-nm-thick channel layer of SY (poly (para-phenylene vinylene) copolymer, Merck OLED Materials GmbH) was deposited as an emissive channel layer by spin coating. Where necessary, a 20-nm-thick layer of PEDOT:PSS (CLEVIOS^TM^ 4083, H. C. Starck Inc.) was also deposited as a HIL over the EC-cleaned SLG source by spin coating (SLG_3_) prior to the deposition of the SY layer. Subsequently, a 2-nm-thick electron injection layer (EIL) of CsF and a drain electrode of Al (80 nm thick) were deposited on the top of the SY layer in sequence via thermal deposition at a rate of 0.05 nm/s under a base pressure of less than 2.7 × 10^−4^ Pa. The fabricated device was finally encapsulated with an epoxy resin and a glass coverslip in a nitrogen-filled glove box.

### Characterisations of the SLGs and SLG-based devices

The variations in the surface roughness and surface potential of the SLG on the substrate were monitored using non-contact AFM and simultaneous KPFM (FlexAFM, Nanosurf AG), respectively, by applying an AC voltage of 1 V at a frequency of 18 kHz to a Pt/Ir-coated silicon tip. To calibrate the work function of the SLG studied here, highly oriented pyrolytic graphite (HOPG, ZYB, Optigraph GmbH) was used as a reference surface. The microscopic morphology of the device was observed by field emission scanning electron microscopy (SEM, Model JSM-6700F, JEOL Co.).

The device performance of the Gr-VOLET was measured using a chroma meter (CS-2000, Konica Minolta) in conjunction with two source meters (2636 A, Keithley). The emission characteristics of the devices were also investigated using an LED measurement system (LCS-100, SphereOptics Inc.) with an integrating sphere. For the operation of the Gr-VOLETs, source-drain voltage *V*_SD_ ( = −*V*_DS_) on the Al drain and gate voltage, *V*_GS_, were applied with respect to the SLG source electrode, held at ground potential.

## Supplementary information

Supplementary Video S1

Supplementary Information
