# Supplementary material for: Full-surface emission of graphene-based vertical-type organic light-emitting transistors with high on/off contrast ratios and enhanced efficiencies
Source: Sci Rep. 2019 Apr 19;9:6328. doi: 10.1038/s41598-019-42800-y (PMC6474894; doi:10.1038/s41598-019-42800-y)
Supplement: Supplementary file 2 — Supplementary Information [file 41598_2019_42800_MOESM2_ESM.pdf]

SUPPLEMENTARY INFORMATION

Full-surface emission of graphene-based vertical-type organic light-emitting transistors with high on/off contrast ratios and enhanced efficiencies

Byoungchoo Park*, Won Seok Lee, Seo Yeong Na, Jun Nyeong Huh, and In-Gon Bae

Department of Electrical and Biological Physics, Kwangwoon University,

Wolgye-Dong, Nowon-gu, Seoul 139-701, Republic of Korea

*e-mail: bcpark@kw.ac.kr

(Supplementary Information)

A. Fabrication steps and light emission of Gr-VOLETs

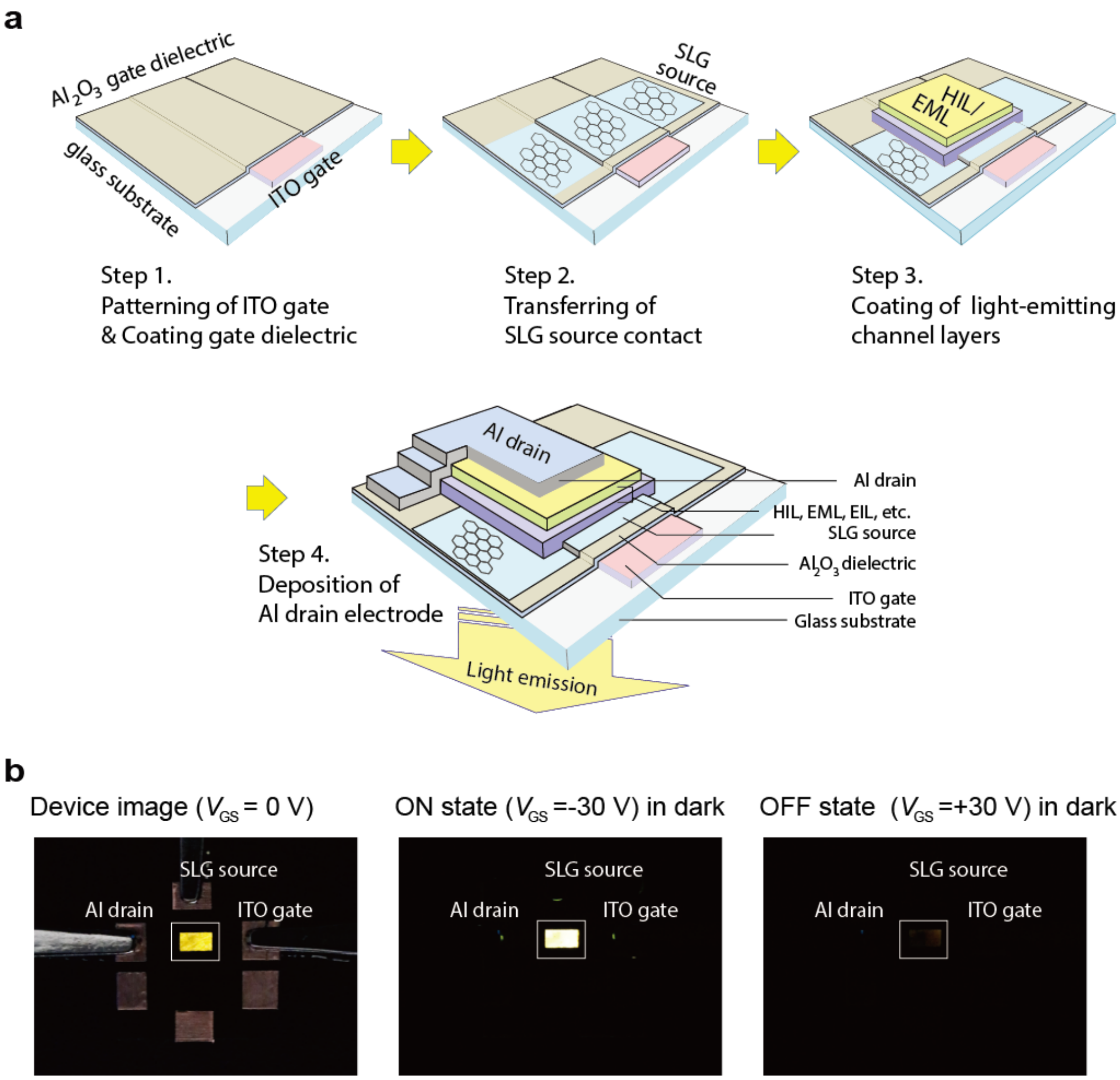

Figure S1. Fabrication steps and light emission of Gr-VOLETs with SLG sources. (**a**) Structure and fabrication steps of graphene-based vertical organic light-emitting transistors (Gr-VOLETs). (**b**) Light emission from a Gr-VOLET (4 mm × 2 mm, white squares) in the dark for the ON and OFF states at different gate voltages V_{GS}s (-30 V and + 30V) at a fixed source-drain voltage V_{SD} of 3.8 V.

Supplementary Fig. S1a presents a schematic illustration of the structure and stages of fabrication of our graphene-based vertical organic light-emitting transistors (Gr-VOLETs) with a single-layer graphene (SLG) source, stacked organic functional channel layers, an Al drain, and an ITO gate separated by an Al_2O_3 gate dielectric layer. The optical characteristics of the VOLET substrate were investigated using a UV-visible spectroscopy system (8453, Agilent). The average optical transmittance (~92%) of a SLG source on a VOLET substrate in the visible range (400~800 nm) was found to be similar to that (~92%) of a conventional ITO-coated glass substrate for OLEDs.

Supplementary Fig. S1b shows the light emission from a Gr-VOLET (4 mm × 2 mm, white squares) for the ON and OFF states at different gate voltages V_{GS}s (-30 V and + 30V) at a fixed source-drain voltage V_{SD} of 3.8 V in the dark taken in order to judge the on/off ratio of the VOLET brightness..

B. Characteristics of CVD graphene

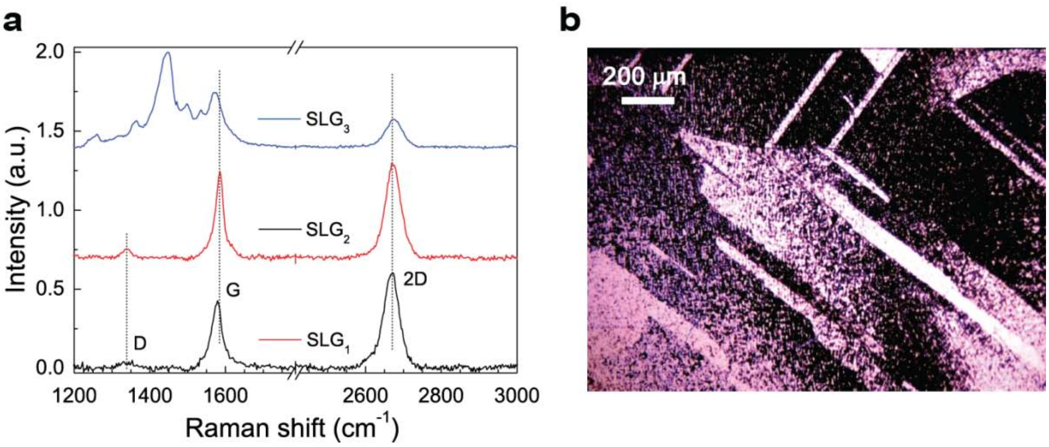

Figure S2. Characteristics of CVD graphene transferred on to VOLET substrates
a Raman spectra of the SLGs transferred from the Cu foil on to the VOLET substrates. **b** Polarised optical microscope image of a spin-coated layer of commercial nematic liquid crystals on SLG_1 transferred to the VOLET substrate.

For the SLGs studied, Raman spectroscopy was carried out using a confocal Raman system (LabRam Aramis, Horiba Jobin-Yvon) with a laser source operating at 514.5 nm (~1 mW on sample). As shown in Supplementary Fig. S2a, the Raman spectra of the SLGs studied have two strong characteristic peaks, a G band at around ~1580~1600 cm^{-1}, which is due to the E_{2g} vibrational mode of sp^2-bonded carbon atoms, and a 2D band at around ~2644~2665 cm^{-1}, which is a second-order type of vibration caused by the scattering of phonons at the zone boundary[1,2]. There are very small disorder-induced

D bands around ~1340~1350 cm^{-1}, indicating the sparse formation of sp^3 bonds due to fewer defects in the SLGs studied.

From the Raman peak intensities, it was found that the ratios of the integrated intensities of the G band to the 2D band for the $FeCl_3$-doped SLG (SLG_1) and the cleaned SLG (SLG_2) sources were in the approximate range of 1.7~1.8, indicating that the SLGs studied here are high-quality monolayer graphenes[2]. Moreover, from the peak positions, it was found that while the G and 2D peaks of SLG_2 are at ~1579 cm^{-1} and ~2669 cm^{-1}, respectively, the G and 2D peak positions of SLG_1 are correspondingly upshifted to ~1585 cm^{-1} and ~2677 cm^{-1}. Similar to SLG_1, it was found that the PEDOT:PSS HIL-coated SLG (SLG_3) has a G peak at ~1585 cm^{-1} and a 2D peak at ~2674 cm^{-1}. By comparison of these with other examples in an earlier report on the relationship between the G and 2D peak positions of graphenes[2], it was verified that SLG_2 is a type of pristine graphene, whereas SLG_1 and SLG_3 are p-type doped graphenes.

The densities of defects, distance between defects, and porosities of nano-defects for both SLG_1 and SLG_2 were estimated from the ratios of the intensities of the G bands to the D bands, I_D/I_G, as shown in the above Raman spectra. The density

of defects (n_D) and distance between defects (L_D) for SLG_1, estimated by the carbon amorphization trajectory ($I_D/I_G \sim 0.117$)[3,4], were $n_D \sim 3.0 \times 10^{10}$ /cm^2 and $L_D \sim 32.8$ nm, respectively, corresponding to a porosity of 9.4×10^{-2} %. Similar to SLG_1, it was found that the SLG_2 has $I_D/I_G \sim 0.113$ and thus n_D of 2.9×10^{10} cm^{-2} and $L_D \sim 33.4$ nm, corresponding to a porosity of 9.1×10^{-2} %. This result clearly indicates that the SLGs studied here are nonporous high-quality graphenes with a negligible number of porous defects introduced during synthesis, transfer, and EC cleaning treatment.

Next, for the SLG_1 studied, polarised optical microscopy was also carried out using SLG_1 covered with commercial nematic liquid crystals (NLCs, Merck LC ZLI-2293) under a crossed polarisation state[5]. As shown in Supplementary Fig. S2b, the polarised optical microscopic image of a spin-coated NLC layer on SLG_1 shows large graphene domains (with an average radius of the domains > 100 μm) in the form of highly uniform optical retardation, beside small domains of several hundred nanometers[6,7], clearly indicating that the SLGs studied here are high-quality graphenes with large-area graphene domains.

C. Basic characteristics of SLGs used in Gr-VOLETs

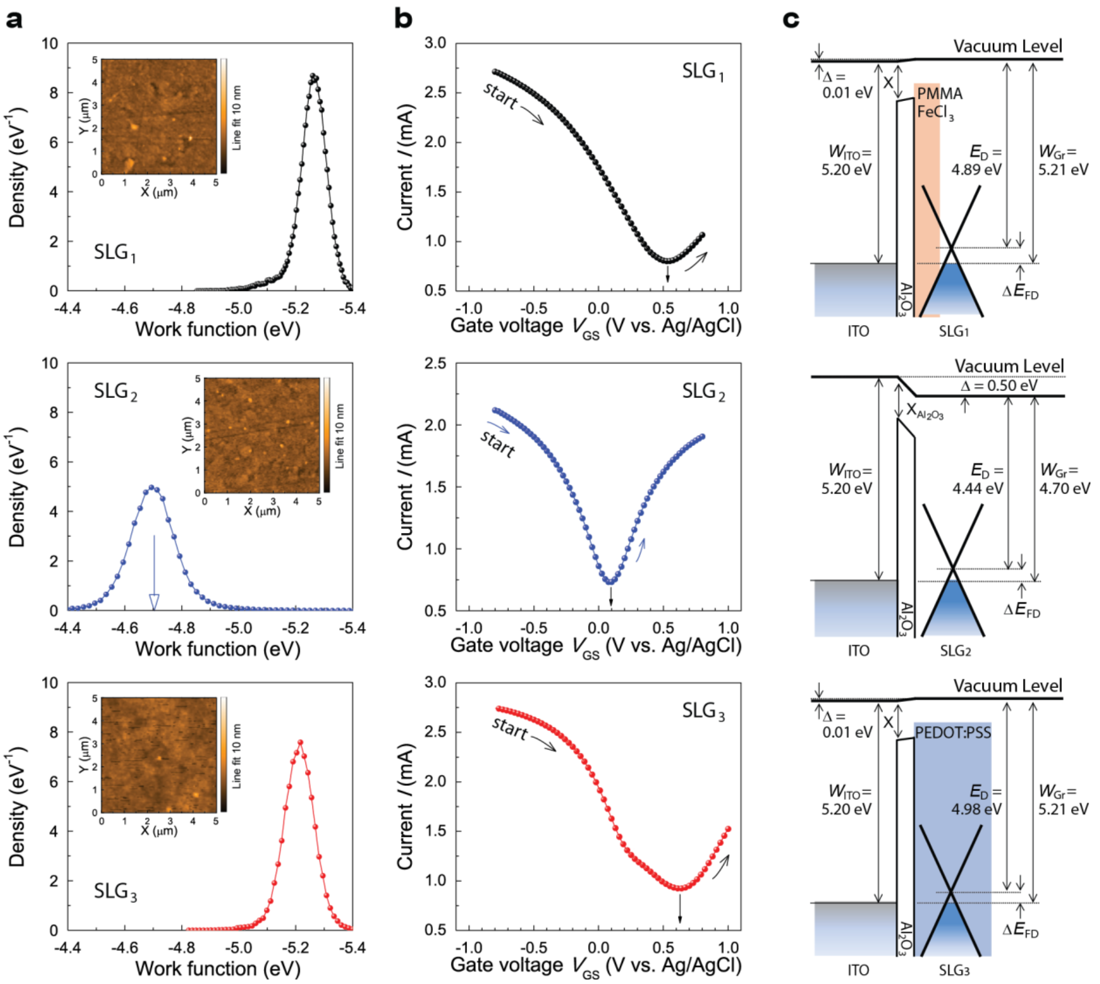

Figure S3. Characteristics of the SLGs used in the Gr-VOLETs. a Work function distributions of the three SLGs on the VOLET substrates as measured by KPFM. The insets show their corresponding AFM topographic images (5 μm × 5 μm). **b** Transport characteristics of the SLGs from liquid-gated lateral Gr-FETs at V_{DS} = -100 mV. **c** Schematic energy band diagrams of graphene Fermi-Dirac cones of the SLGs on the VOLET substrates at V_{GS} = 0 V. W: work function, E_D: Dirac point energy of the SLG, X: electron affinity of Al_2O_3 (~1.0 eV), and ΔE_{FD}: Fermi level (E_F) with reference to the Dirac point energy (E_D).

Supplementary Fig. S3 presents the basic electronic properties of the studied SLGs on the VOLET substrates. Supplementary Fig. S3a shows the distributions of the work functions of the SLGs as measured by KPFM. The work function of the SLG (W_{SLG}) was obtained by a comparison of the surface contact potential differences (V_{CPD}s) measured by KPFM for the SLG and the HOPG, *i.e.*, $W_{SLG} = W_{HOPG} + [V_{CPD}(HOPG) - V_{CPD}(SLG)]$, where W_{HOPG} is the work function of HOPG (~4.6 eV)[8,9]. While the estimated average work function of the cleaned SLG (SLG_2) on the VOLET substrate is approximately 4.70±0.10 eV, which is in reasonably good agreement with the intrinsic work function (4.5~4.8 eV) of monolayer graphenes (middle panel)[9,10], the work function of the $FeCl_3$-doped SLG (SLG_1) is increased to 5.21±0.07 eV (upper panel), mainly due to $FeCl_3$ doping[11]. Similar to SLG_1, the estimated work function of the PEDOT:PSS HIL-coated SLG (SLG_3) is approximately 5.21±0.06 eV (lower panel). We also investigated the AFM morphologies of the SLGs (insets in Supplementary Fig. S3a). As shown by the AFM morphologies, the SLGs exhibit fairly smooth surfaces on the VOLET substrates; the SLGs present AFM morphologies that are nearly identical, with a low RMS roughness of 1.4~2.0 nm.

Next, the transport characteristics of the SLGs used were observed by assessing liquid-gated lateral Gr-FETs with SLG channels (channel length 50 μm, Supplementary Fig. S4), as shown in Supplementary Fig. S3b. For the SLG_1 channel, the Gr-FET shows a clear asymmetrical V-shaped I_D-V_{GS} curve with a charge-neutral gate voltage (or Dirac point, V_{Dirac}) of ~0.54 $V/V_{Ag/AgCl}$. This large positive value of V_{Dirac} clearly indicates that SLG_1 is p-type (hole) doped graphene due to the chlorination of graphene by $FeCl_3$ (upper panel in Supplementary Fig. S3b)[11]. According to the V_{Dirac} of SLG_1, the energy level of the Dirac point E_D, relative to the vacuum level, can be estimated with respect to the redox potential of a probe material of ferrocene via the relationship: $E_D = [-(e\ V_{G,Dirac} - E_{1/2(Fc/Fc+)}) - 4.8]$ eV. Here, 4.8 eV is the absolute energy level of the ferrocene/ferrocenium (Fc/Fc+) redox couple below the vacuum level and $E_{1/2(Fc/Fc+)} = 0.45$ eV [12]. From the above relationship, the V_{Dirac} value of ~0.54 $V/V_{Ag/AgCl}$ for SLG_1 gives a Dirac point energy E_D of approximately ~4.89 eV. Note that the E_D value of 4.89 eV is much higher than that (~4.49 eV) of monolayer epitaxial graphene[13], also confirming the p-type doping of the SLG_1. In contrast to SLG_1, the SLG_2 channel showed clear, symmetrical V-shaped transfer characteristics with a Dirac point (V_{Dirac} ~ 0.09 $V/V_{Ag/AgCl}$) (middle panel in Supplementary Fig. S3b), confirming that SLG_2 is undoped and intrinsic SLG. From the $V_{G,Dirac}$ value of SLG_2,

the estimated E_D is approximately ~4.44 eV. Note that the E_D value of 4.44 eV is in good agreement with that (~4.49 eV) of epitaxial monolayer graphene[13]. which again confirms that the SLG_2 source used here is certainly undoped and intrinsic SLG. Similar to SLG_1, the SLG_3 channel also shows a clear, asymmetrical V-shaped curve with a V_{Dirac} value of ~0.63 V/V $_{Ag/AgCl}$ (lower panel in Supplementary Fig. S3b). With this V_{Dirac}, the estimated value of E_D for SLG_3 is approximately ~4.98 eV. Thus it is noted that the work function and Dirac point energy of SLG_3 are similar to those of SLG_1, indicating that the PEDOT:PSS HIL may have a p-type doping effect on SLG[11]. From the transfer characteristics, the carrier mobilities μ of the SLGs were also estimated using the relationship $\mu = (L/WC_g V_{DS})(\Delta I_D/\Delta V_{GS})$ [14], where L, W, and C_g are respectively the channel length (50 μm), the width (1600 μm), and the top-gate capacitance of graphene (~1.9 μF/cm^2) [15]. The estimated hole mobilities for SLG_1 and SLG_3 are approximately ~410 cm^2/(V s) and ~530 cm^2/(V s), respectively, while the hole and electron mobilities for SLG_2 are approximately ~580 cm^2/(V s) and ~530 cm^2/(V s), respectively (Table 1).

The above observations allow us to deduce the energy band diagrams of the studied SLGs on VOLET substrates at $V_{GS} = 0$ V (Supplementary Fig. S3c). In the diagrams, ΔE_{FD} represents the Fermi level with respect to its Dirac point energy E_D. For

SLG$_1$, ΔE_{FD} is approximately 0.32 eV, which is slightly higher than those of both SLG$_2$ (0.26 eV) and SLG$_3$ (0.23 eV). The potential difference (Δ) with regard to the ITO/Al_2O_3/SLG interfaces was also determined; the Δ values are -0.01 eV for SLG$_1$, 0.50 eV for SLG$_2$, and -0.01 eV for SLG$_3$, showing significant reductions in the potential differences at the interfaces after doping the SLGs.

D. Device structures of lateral FET substrate and liquid-gated lateral Gr-FET

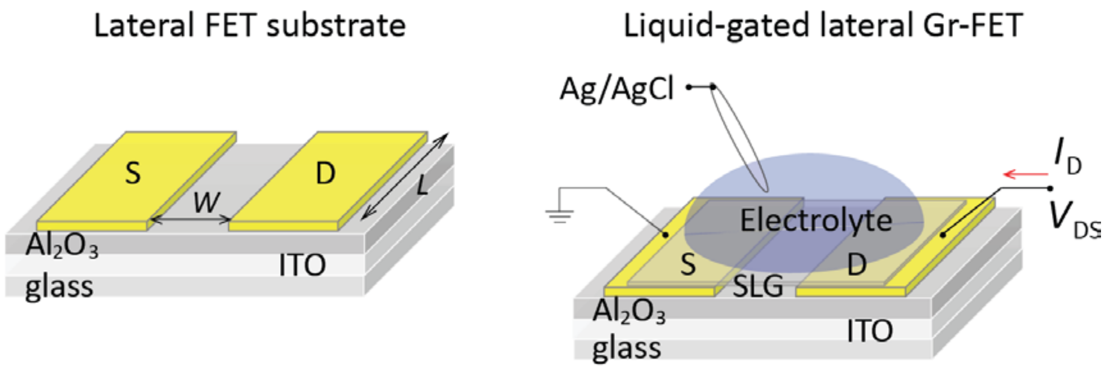

Figure S4. Lateral FET substrate and liquid-gated lateral Gr-FET. Left: the structure of the lateral FET substrate. (*L*: channel length, 50 μm, *W*: channel width, 1600 μm). Right: the structure of the liquid-gated lateral Gr-FET with an Ag/AgCl reference electrode in a non-aqueous electrolyte containing ACN and 100 mM of $TBAPF_6$.

Regarding the transport characteristics of the SLGs studied, a liquid-gated lateral graphene FET (Gr-FET) was prepared using an FET substrate with the ACN electrolyte,

which was identical to that used in the EC-cleaning treatment (see Methods section). The channel of the studied SLG of the Gr-FET was gated through the ACN electrolyte with the Ag/AgCl reference electrode by sweeping the gate voltages from -0.8 to 0 V and then to +0.8 V with a sweep rate of 30 mV/s at V_{DS} = 100 mV. In general, the liquid-gate Gr-FET has better transfer characteristics than conventional back-gate Gr-FETs, because the liquid gate exhibits higher capacitance than the back gate[11,15]. The electrical characteristics of the Gr-FETs were measured using a source meter (Keithley 2400).

E. Output characteristics of Gr-VOLETs with reference SLG sources and comparison with their control ITO-OLEDs

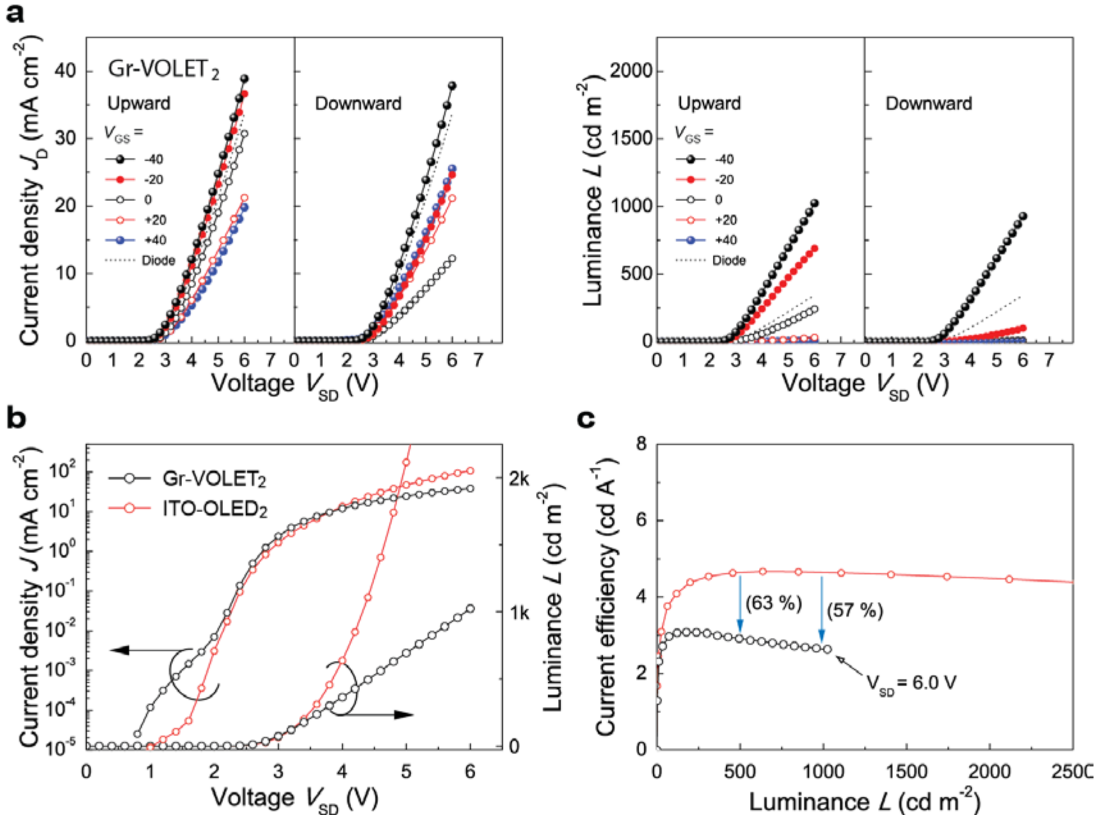

Figure S5. Output characteristics of Gr-VOLET$_2$ and comparison with the control ITO-OLED$_2$. a Gate-voltage (V_{GS})-dependent current density-voltage (J_D-V_{SD}) (left) and luminance-voltage (L-V_{SD}) (right) characteristics of Gr-VOLET$_2$ with a pristine SLG$_2$ source for upward and downward changes of V_{GS}. For comparison, the characteristics of a gate-disconnected Gr-VOLET$_2$ (*i.e.*, Gr-OLED$_2$) are also shown (dotted curves, OLED operations). J-L-V (**b**) and η_C-L (**c**) comparisons of Gr-VOLET$_2$ in the bright on-state (V_{GS} = −40 V) with its respective ITO-based control OLED (ITO-OLED$_2$). Note that ITO-OLED$_1$ = ITO-OLED$_2$ = (ITO/SY/CsF/Al).

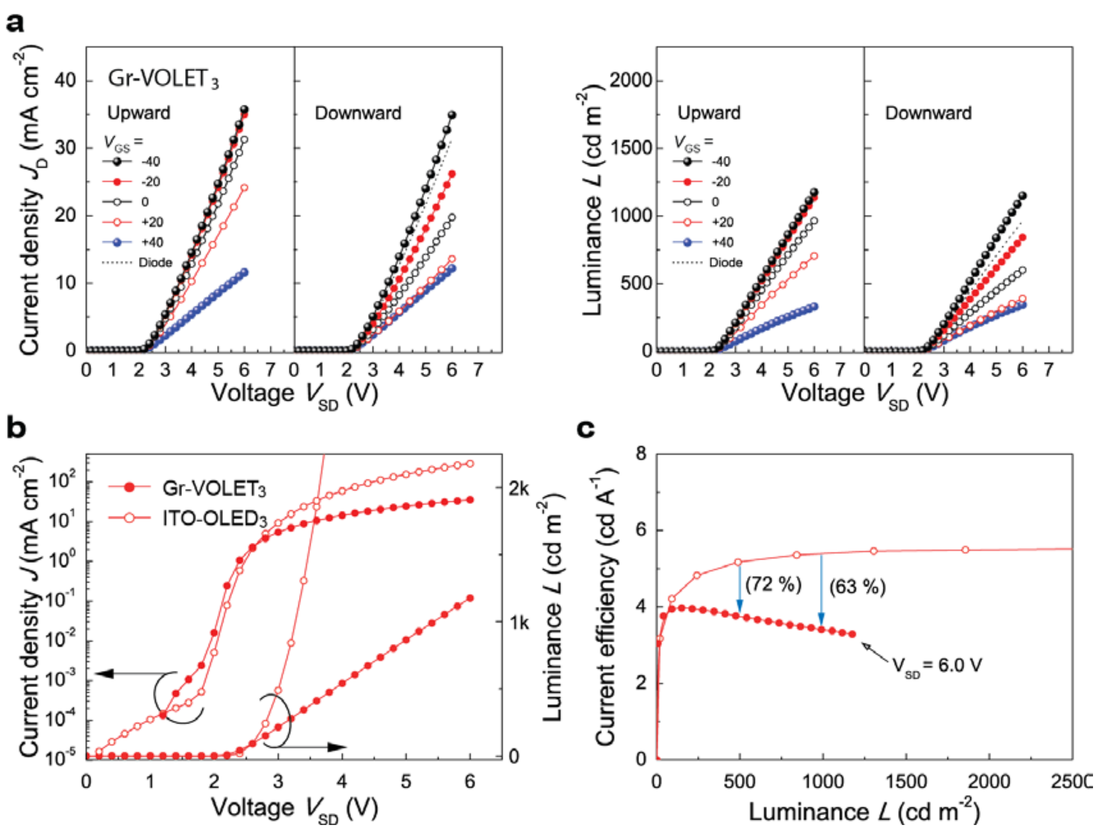

Figure S6. Output characteristics of Gr-VOLET$_3$ and comparison with the control ITO-OLED$_3$. a Gate-voltage (V_{GS})-dependent current density-voltage (J_D-V_{SD}) (left) and luminance-voltage (L-V_{SD}) (right) characteristics of Gr-VOLET$_3$ with a PEDOT:PSS HIL-coated SLG$_3$ source for upward and downward changes of V_{GS}. For comparison, the characteristics of a gate-disconnected Gr-VOLET$_3$ (*i.e.*, Gr-OLED$_3$) are also shown (dotted curves, OLED operations). *J*-*L*-*V* (**b**) and η_C-L (**c**) comparisons of Gr-VOLET$_3$ in the bright on-state (V_{GS} = −40 V) with its respective ITO-based control OLED (ITO-OLED$_3$). Note that ITO-OLED$_3$ = (ITO/PEDOT:PSS/SY/CsF/Al).

F. Temperature dependence of electrical characteristics for Gr-VOLET

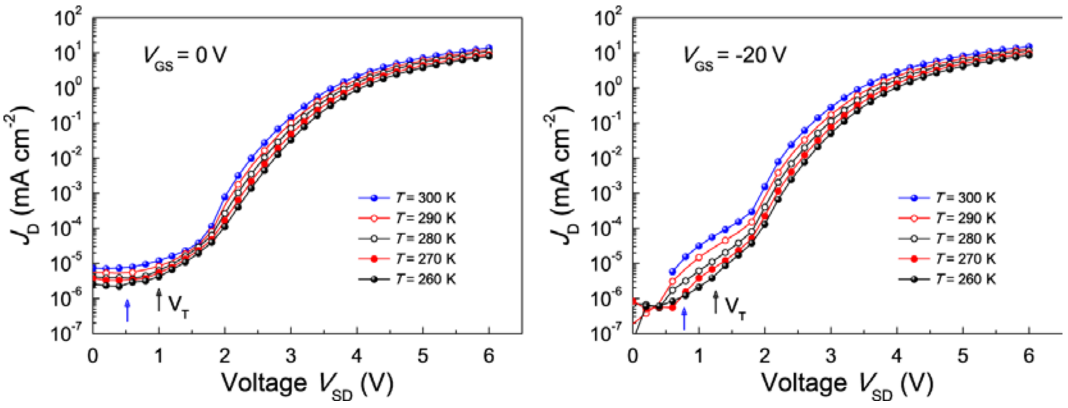

Figure S7. Temperature dependence of the electrical characteristics for the Gr-VOLET. J_D- V_{SD} characteristics of a Gr-VOLET with a $FeCl_3$-doped SLG source at V_{GS} = 0 V (left) and at V_{GS} = -20 V (right) for five different temperatures. These characteristic curves indicate that the charge injection at the SLG_1/SY interface has little temperature dependence, especially for V_{SD} > V_T (~0.6~1.3 V), for the temperature range investigated.

G. Characteristics of TIPS-PEN OTFT used

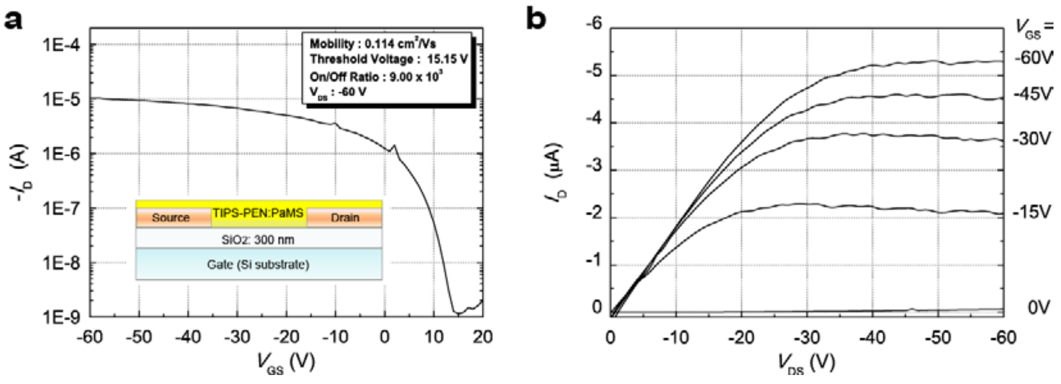

Figure S8. Characteristics of TIPS-PEN OTFT used. Transfer curves (**a**) and output series (**b**) measured for the TIPSPEN:PaMS OTFT device used here. The inset shows a schematic of the TIPS-PEN:PaMS OTFT.

For bottom-contact OTFT devices, we used a SiO_2/Si FET substrate, as described in the Methods Section. Prior to the deposition of the active layer, the substrates were cleaned using UV ozone. A self-assembled monolayer of pentafluorobenzenethiol (Aldrich) was formed on the Au electrodes to improve the metal/organic contact. Hexamethyldisilazane (Aldrich) was then spin-coated on the substrate at 4000 rpm and baked at 125 °C for 10 min. The active channel layer was then solution-coated on the substrates from a blended solution using a simple solution-coating method[16]. The blended solution was a mixture of 6,13-bis(triisopropylsilylethynyl)-pentacene (TIPS-PEN, Sigma Aldrich) and the polymer binder poly(a-methylstyrene) (PaMS, Sigma Aldrich) (0.3:0.7 wt%), which were dissolved in toluene. The coated active layers were then dried at 100 °C for 60 min using a heating plate, in order to remove the remaining solvents. Then, the transfer and output characteristics of the fabricated TIPS-PEN:PaMS OTFT were recorded using a semiconductor parametric analyser with a source meter (Keithley 2400). Supplementary Fig. S8 shows representative examples of log (I_D) vs. V_{GS} at V_{DS} = -60 V (Supplementary Fig. S8a) and I_D vs. V_{DS} for several

gate voltages (Supplementary Fig. S8b) for TIPS-PEN:PaMS OTFTs. The OTFT exhibited a mobility of 0.11 $cm^2/(V\ s)$, a threshold voltage of 15.2 V, and an on/off current ratio of 0.9×10^4 at a V_{DS} of -60 V. This value of the mobility of the OTFT is similar to that (0.16~0.20 $cm^2/(V\ s)$) quoted in previous reports[16].

I. Supplementary Video

Supplementary Video S1: Our Gr-VOLET (4 mm × 2 mm) with a $FeCl_3$-doped SLG source in operation.

J. Supplementary References

1. Ferrari, A. C. Raman spectroscopy of graphene and graphite: Disorder, electron–phonon coupling, doping and nonadiabatic effects. *Solid State Commun.* **143**, 47-57 (2007).
2. Wang, Q. H., Jin, Z., Kim, K. K., Hilmer, A. J., Paulus, G. L. C., Shih, C.-J., Ham, M.-H., Sanchez-Yamagishi, J. D., Watanabe, K., Taniguchi, T., Kong, J., Jarillo-Herrero, P. & Strano, M. S. Understanding and controlling the substrate effect on graphene electron-transfer chemistry via reactivity imprint lithography. *Nat. Chem.* **4**, 724-732 (2012).
3. Cançado, L. G., Jorio, A., Martins Ferreira, E. H., Stavale, F., Achete, C. A., Capaz, R. B., Moutinho, M. V. O., Lombardo, A., Kulmala, T. S. & Ferrari, A. C. Quantifying Defects in Graphene via Raman Spectroscopy at Different Excitation Energies. *Nano Lett.* **11**, 3190-3196 (2011).
4. Huang, S., Dakhchoune, M., Luo, W., Oveisi, E., He, G., Rezaei, M., Zhao, J., Alexander, D. T. L., Züttel, A., Strano, M. S. & Agrawal, K. V. Single-layer graphene membranes by crack-free transfer for gas mixture separation. *Nat. Commun.* **9**, 2632 (2018).

5. Kim, D. W., Kim, Y. H., Jeong, H. S. & Jung, H.-T. Direct visualization of large-area graphene domains and boundaries by optical birefringency. *Nat. Nanotechnol.* **7**, 29-34 (2012).
6. Huang, P. Y., Ruiz-Vargas, C. S., van der Zande, A. M., Whitney, W. S., Levendorf, M. P., Kevek, J. W., Garg, S., Alden, J. S., Hustedt, C. J., Zhu, Y., Park, J., McEuen, P. L. & Mulle, D. A. Grains and grain boundaries in single-layer graphene atomic patchwork quilts. *Nature* **469**, 389-392 (2011).
7. Nemes-Incze, P., Yoo, K. J., Tapasztó, L., Dobrik, G., Lábár, J., Horváth, Z. E., Hwang, C. & Biró, L. P. Revealing the grain structure of graphene grown by chemical vapor deposition. *Appl. Phys. Lett.* **99**, 023104 (2011).
8. Takahashi, T., Tokailin, H. & Sagawa, T. Angle-resolved ultraviolet photoelectron spectroscopy of the unoccupied band structure of graphite. *Phys. Rev. B* **32**, 8317-8324 (1985).
9. Yu, Y.-J., Zhao, Y., Ryu, S., Brus, L. E., Kim, K. S. & Kim, P. Tuning the Graphene Work Function by Electric Field Effect. *Nano Lett.* **9**, 3430-3434 (2009).
10. Panchal, V., Pearce, R., Yakimova, R., Tzalenchuk, A. & Kazakova, O. Standardization of surface potential measurements of graphene domains. *Sci. Rep.* **3**, 2597 (2013).
11. Park, B., Huh, J. N., Lee, W. S. & Bae, I.-G. Simple and rapid cleaning of graphenes with a 'bubble-free' electrochemical treatment. *J. Mater. Chem. C* **6**, 2234-2244 (2018).
12. Padhy, H., Huang, J.-H., Sahu, D., Patra, D., Kekuda, D., Chu, C.-W. & Lin, H.-C. Synthesis and Applications of Low-Bandgap Conjugated Polymers Containing Phenothiazine Donor and Various Benzodiazole Acceptors for Polymer Solar Cells. *J. Polym. Sci. A Polym. Chem.* **48**, 4823-4834 (2010).
13. Gugel, D., Niesner, D., Eickhoff, C., Wagner, S., Weinelt, M. & Fauster, T. Two-photon photoemission from image-potential states of epitaxial graphene. *2D Mater.* **2**, 045001 (2015).
14. Schwierz, F. Graphene transistors. *Nat. Nanotechnol.* **5**, 487-496 (2010).
15. Ohno, Y., Maehashi, K., Yamashiro, Y. & Matsumoto, K. Electrolyte-Gated Graphene Field-Effect Transistors for Detecting pH and Protein Adsorption. *Nano Lett.* **9**, 3318-3322 (2009).
16. Park, B., Jeon, H. G., Choi, J., Kim, Y. K., Lim, J., Jung, J., Cho, S. Y. & Lee, C. High-performance organic thin-film transistors with polymer-blended small-molecular semiconductor films, fabricated using a pre-metered coating process. *J. Mater. Chem.* **22**, 5641-5646 (2012).
